# Shaping plant architecture

**DOI:** 10.3389/fpls.2015.00233

**Published:** 2015-04-09

**Authors:** Thomas Teichmann, Merlin Muhr

**Affiliations:** Plant Cell Biology, Georg-August-Universität Göttingen, GöttingenGermany

**Keywords:** axillary meristem, branching, apical dominance, auxin, cytokinins, strigolactone, ideal plant architecture, poplar

## Abstract

Plants exhibit phenotypical plasticity. Their general body plan is genetically determined, but plant architecture and branching patterns are variable and can be adjusted to the prevailing environmental conditions. The modular design of the plant facilitates such morphological adaptations. The prerequisite for the formation of a branch is the initiation of an axillary meristem. Here, we review the current knowledge about this process. After its establishment, the meristem can develop into a bud which can either become dormant or grow out and form a branch. Many endogenous factors, such as photoassimilate availability, and exogenous factors like nutrient availability or shading, have to be integrated in the decision whether a branch is formed. The underlying regulatory network is complex and involves phytohormones and transcription factors. The hormone auxin is derived from the shoot apex and inhibits bud outgrowth indirectly in a process termed apical dominance. Strigolactones appear to modulate apical dominance by modification of auxin fluxes. Furthermore, the transcription factor BRANCHED1 plays a central role. The exact interplay of all these factors still remains obscure and there are alternative models. We discuss recent findings in the field along with the major models. Plant architecture is economically significant because it affects important traits of crop and ornamental plants, as well as trees cultivated in forestry or on short rotation coppices. As a consequence, plant architecture has been modified during plant domestication. Research revealed that only few key genes have been the target of selection during plant domestication and in breeding programs. Here, we discuss such findings on the basis of various examples. Architectural ideotypes that provide advantages for crop plant management and yield are described. We also outline the potential of breeding and biotechnological approaches to further modify and improve plant architecture for economic needs.

## Introduction

**FIGURE 1:**
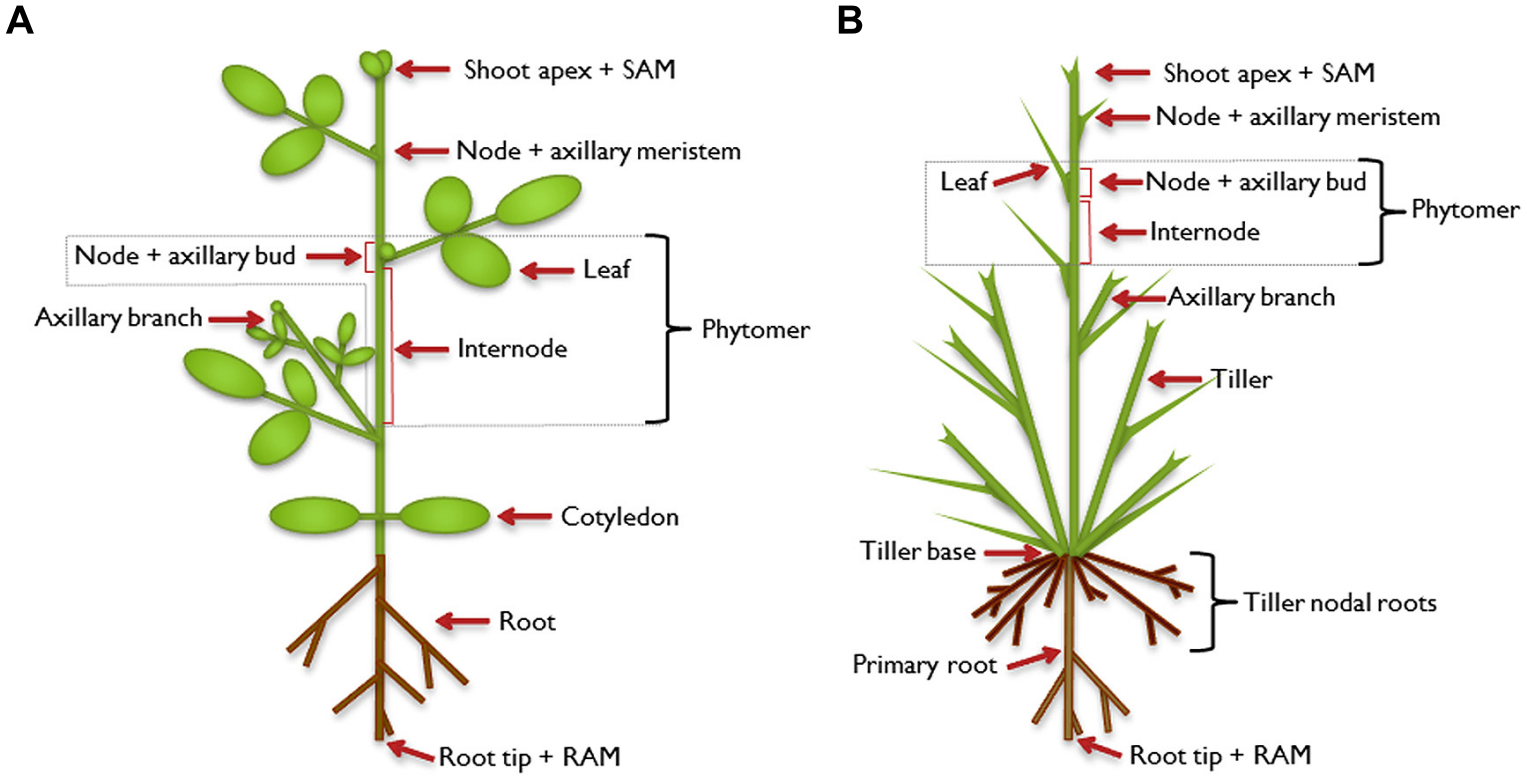
**Illustration of plant architecture**. Typical architecture of a dicot plant **(A)** and a monocot plant **(B)**. The shoot apical meristem (SAM) establishes the shoot as the primary growth axis of the plant by continuously initiating phytomers, the basic modules of the plant shoot. A phytomer consists of an internode and a node with its attached leaf. In the leaf axils, axillary (secondary) meristems are formed in dicot and some monocot plants, which develop into an axillary bud and have the potential to continue growth to form an axillary branch. This branch can be regarded as a secondary growth axis and is built in the same way as the primary shoot. It can branch further to form higher-order branches (not shown). The primary root is established by its own meristem [root apical meristem (RAM)] and can also branch to form secondary or higher-order lateral roots. In addition to axillary branches, monocot plants can produce tillers which emanate from the base of the plant, which has extremely condensed internodes. The tillers form adventitious roots, called tiller nodal roots.

As sessile organisms, plants cannot escape from their habitat. They have to cope with the prevailing conditions, including abiotic factors like nutrient supply and biotic influences such as herbivory. Part of the adaptation strategy toward those challenges is an enormous degree of flexibility in plant architecture which is facilitated by the open, indeterminate development of plants. During plant embryogenesis, the apical-basal axis is established. At the poles of this axis, shoot and root apical meristems (SAM and RAM), respectively, develop as primary meristems. With the onset of post-embryonic development, the SAM extends the primary growth axis of the above-ground part of the plant. So-called phytomers are formed as repetitive basal modules of the plant shoot which consist of an internode and a node with one or more attached axillary leaves (**Figure 1**). In the leaf axils, secondary, lateral meristems are established and allow the formation of higher order morphological structures. The axillary meristems may develop a bud that can extend to form a branch, which constitutes a secondary growth axis. Branches are built up in the same way as the primary growth axis, and higher order branching can occur, leading to a complex structure. The architecture of a mature plant is therefore determined by the number and activity of axillary meristems and the growth characteristics of the branches that develop from axillary buds (Kerstetter and Hake, 1997; Sussex and Kerk, 2001; McSteen and Leyser, 2005; Schmitz and Theres, 2005; Bennett and Leyser, 2006; De Smet and Juergens, 2007; Janssen et al., 2014).

## Axillary Meristem Initiation

Axillary meristems are the origin of lateral branches. They are formed in the center of the boundary zone at the adaxial side of the leaf base. The boundary zone separates the shoot apical meristem (SAM) from the developing leaf primordium. This zone is not just a border but fulfills an important function in meristem maintenance and organ development (Zadnikova and Simon, 2014). It is characterized by small cells, stiff cell walls and a low cell division rate. A key factor during establishment of the boundary zone is the transcription factor LATERAL ORGAN BOUNDARIES1 (LOB1) that induces the expression of *BAS1*, encoding a protein that has brassinosteroid inactivating activity (Bell et al., 2012). Brassinosteroids are plant steroid hormones that influence cell expansion and cell division (reviewed in Hardtke, 2007; Fridman and Savaldi-Goldstein, 2013). The LOB1-mediated decrease of brassinosteroid activity causes a reduction of cell size and cell division rate in the boundary zone compared to neighboring zones (Bell et al., 2012; Gendron et al., 2012). This effect is enhanced by the outward orientation of the auxin eﬄux carrier PIN-FORMED1 (PIN1) causing depletion of the plant growth hormone auxin in the boundary zone. During initial outgrowth of the leaf primordium, PIN1 is oriented toward the primordium. However, as the boundary zone develops, PIN1 is reoriented toward the SAM (Wang et al., 2014a,b). This reorientation depends on the kinase PINOID (PID) that controls basal-apical localization of PIN1 (Furutani et al., 2004). The importance of PIN reorientation and the role of PID in development of a functional boundary zone can be seen in *pin1* and *pid* mutants that exhibit defects in axillary meristem formation (Wang et al., 2014a,b). Artificial increase of auxin in the developing boundary zone by localized expression of the auxin biosynthesis gene *iaaM* in transgenic *Arabidopsis* resulted in the lack of axillary meristems in a portion of the leaf axils (Wang et al., 2014a,b). On the contrary, boundary zone specific expression of a stabilized version of the AUX/IAA protein BODENLOS to reduce auxin signaling in this area resulted in the formation of axillary buds in the axils of cotyledons which was never observed in wild type plants (Wang et al., 2014a). Therefore, a local auxin minimum in the boundary zone appears to be important for axillary meristem formation.

Another gene having an effect on shoot lateral organ development is *RPS10B*, which was found in a suppressor screen of the *more axillary branching2-1* (*max2-1*) mutant. It encodes the ribosomal protein S10e. Stirnberg et al. (2012a) discuss that in the mutant, levels of proteins, which are important for the regulation of auxin distribution and therefore auxin-mediated organ boundary patterning, may be imbalanced. Especially proteins with a high turnover rate, such as the Aux/IAA repressors involved in auxin signaling, may be affected by the ribosomal *rps10b-1* mutation (Stirnberg et al., 2012a). In the same suppressor screen, *FAR-RED ELONGATED HYPOCOTYL3* (*FHY3*) was found. The authors discuss this gene to be potentially involved in the regulation of auxin homeostasis, too (Stirnberg et al., 2012b).

Therefore, there appear to be many factors controlling the precise spatiotemporal auxin distribution during meristem development. In addition to auxin, Wang et al. (2014b) also discuss a role of cytokinin during AM initiation. They report a cytokinin pulse following and being dependent on the establishment of an auxin minimum in the boundary zone of the leaf axil and provide hints for the importance of cytokinin signaling during the establishment of the axillary meristem.

Tissue markers of the boundary zone are the *Arabidopsis* NAM-ATAF1/2-CUC2 (NAC) transcription factors CUP SHAPED COTYLEDONS1, 2, and 3 (CUC1, 2, and 3; Spinelli et al., 2011) that have redundant functions in meristem formation. In tomato, *GOBLET* (*GOB)* was identified as an ortholog of the *CUC* genes (Busch et al., 2011). Expression of these genes is a prerequisite for development of the SAM and the consecutive formation of the boundary zone. *CUC* genes are down-regulated by brassinosteroids. Thus, low brassinosteroid activity in the boundary zone not only reduces cell expansion and division as described above, but also allows the induction of *CUC* genes (Bell et al., 2012; Gendron et al., 2012).

The most pronounced difference between the SAM, the neighboring boundary zone and the developing leaf primordium is that cells in the SAM are kept in an indeterminate, non-differentiated state while cells of the boundary zone and the primordium differentiate. Meristematic identity of the SAM cells is retained by activity of the homeobox class I *KNOX* gene *SHOOT MERISTEMLESS* (*STM*; Long et al., 1996; Long and Barton, 2000). As soon as cells start to differentiate, *STM* is down-regulated by the MYB transcription factor AS1 and the LATERAL ORGAN BOUNDARY DOMAIN (LBD) transcription factor AS2 (Ikezaki et al., 2010). Interestingly, during an early phase of boundary zone development, *STM* continues to be transcribed in all cells of the boundary zone, albeit at a low level (Long and Barton, 2000). This indicates that, for a restricted time period, cells of the boundary zone keep the capacity to return to a meristematic stage. During this developmental phase, the axillary meristem is initiated (Grbic and Bleecker, 2000). A molecular marker of *de novo* axillary meristem formation is the focused and strong expression of *STM* in the center of the boundary zone. In *Arabidopsis*, this focused *STM* expression depends on the presence of the GRAS transcription factor LATERAL SUPPRESSOR (LAS; Greb et al., 2003). Orthologs of *LAS* are *LS* in tomato (Schumacher et al., 1999) and *MONOCULM1* (*MOC1*) in rice (Li et al., 2003). Knockout mutants of *LAS* fail to develop axillary meristems during the vegetative stage (Greb et al., 2003). Keller et al. (2006) suggested that “LAS is required for reacquisition of indeterminate cell fate in axillary cells in the course of AM organization.”

Axillary meristem initiation and development is modulated by several factors that have partially redundant functions. In addition to LAS, the MYB factors REGULATOR OF AXILLARY MERISTEMS1 (RAX1) in *Arabidopsis* (Keller et al., 2006), as well as BLIND (BL) and POTATO LEAF (C) in tomato (Schmitz et al., 2002; Busch et al., 2011), influence axillary meristem development. Another factor is a basic helix-loop-helix (bHLH) protein called REGULATOR OF AXILLARY MERISTEM FORMATION (ROX) in *Arabidopsis* (Yang et al., 2012), LAX PANICLE1 (LAX1) in rice (Komatsu et al., 2001, 2003) and BARREN STALK1 (BA1) in maize (Ritter et al., 2002; Gallavotti et al., 2004).

For the ontogenetic origin of axillary meristems, two theories have been discussed (Sussex and Kerk, 2001). The *de novo* meristem formation theory is based on the fact that in some plant species, e.g., *Arabidopsis*, axillary meristems cannot be detected after leaf initiation by anatomical studies. In contrast, the detached or reserve meristem theory describes the situation in plants like tomato where meristematic cells from the SAM persist in the axils of newly built leaves and then, later during development, form axillary meristems (reviewed in Bennett and Leyser, 2006). However, the studies on LAS, RAX1, and ROX1 show that similar key factors control meristem initiation in plant species that seem to have contrasting mechanisms of meristem development. This indicates that axillary meristems in plants are generally formed by the same process. The fact that the boundary zone that just separated from the SAM continues to show *STM* expression argues for the detached meristem hypothesis. Cells of the boundary zone seem to be kept in a stage that is not fully determinate and, as a consequence, the axillary meristem can be initiated from this pool of cells. In conclusion, these data provide evidence that also in plants like *Arabidopsis*, where the meristem appears at later stages of development, the meristem is not formed *de novo* but built as a detached meristem (Leyser, 2003; Bennett and Leyser, 2006).

Axillary meristems strictly form on the adaxial side of leaf bases. This may be the reason why the transcription factor REVOLUTA (REV), that determines adaxiality, has been described as a further axillary meristem initiation factor (Otsuga et al., 2001). However, its effect on axillary meristem formation may be secondary and the primary function of REV is the control of radial patterning (Emery et al., 2003; Bennett and Leyser, 2006).

## Activity of Apical Meristems and Control of Bud Outgrowth

**FIGURE 2:**
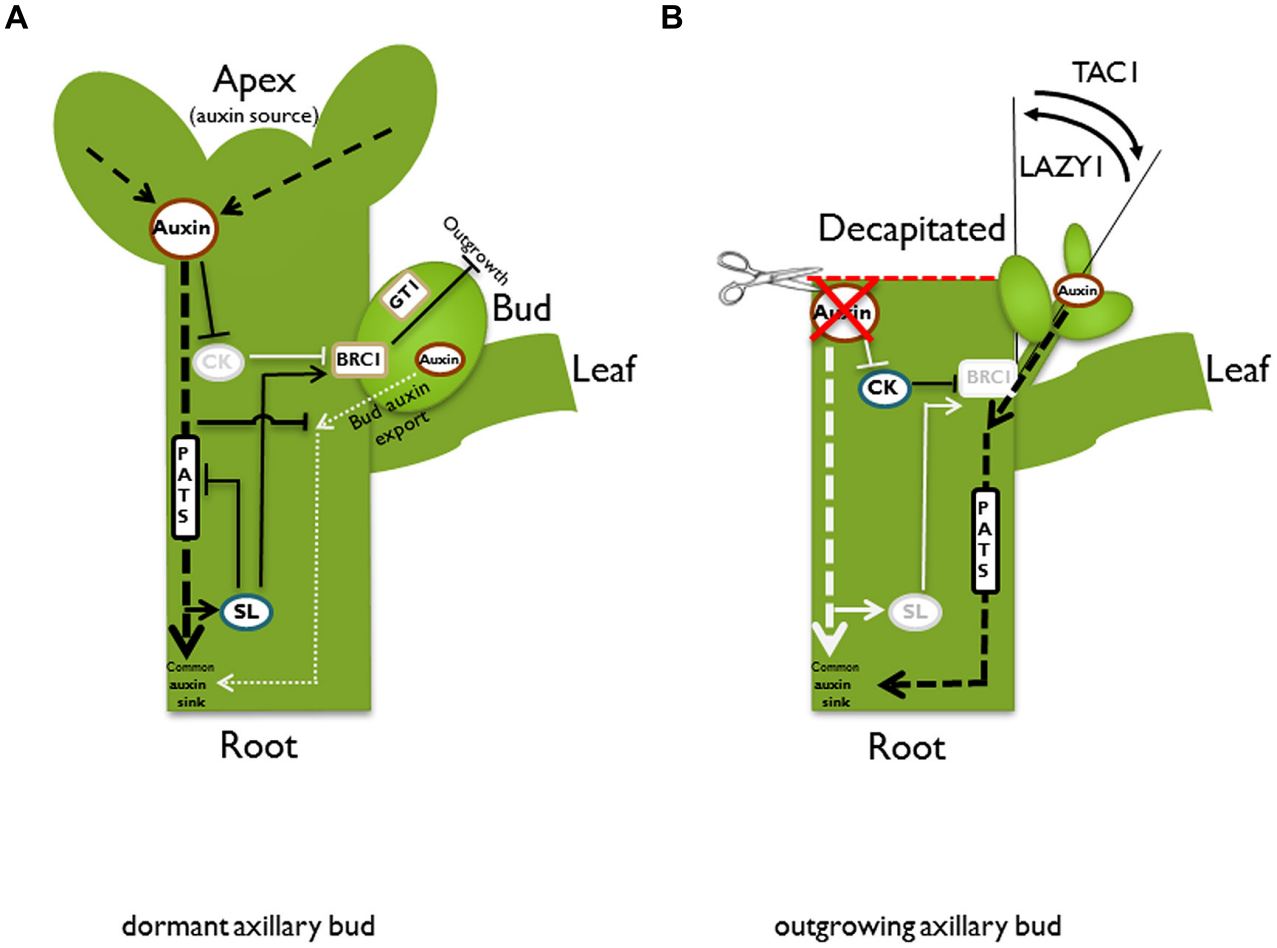
**Schematic illustration of different pathways and models in the control of bud outgrowth**. In an intact plant **(A)**, the apex is a strong auxin source. Auxin is transported basipetally in the polar auxin transport stream (PATS). According to the second messenger model, auxin promotes strigolactone (SL) and represses cytokinin (CK) biosynthesis, respectively. Both hormones have adverse effects on bud outgrowth, most likely acting via the transcription factor *BRANCHED1*/*TEOSINTE BRANCHED1* (*BRC1*/*TB1*). Auxin indirectly promotes *BRC1*/*TB1* expression, which suppresses bud outgrowth. *GRASSY TILLERS1* (*GT1*) is a putative downstream target for *TB1* in monocots. According to the auxin transport canalization model, the axillary bud is also an auxin source and as a prerequisite for vascular tissue formation and bud outgrowth, it has to establish its own auxin export. However, it competes with the shoot apex for the stem as a shared auxin sink. This competition is enhanced by SL, which reduces plasma membrane accumulation of the *PIN1* auxin eﬄux carrier and therefore inhibits the PATS in the main stem. High auxin levels in the stem prevent the formation of an initial auxin export flux from the bud, and therefore suppress bud outgrowth. After decapitation **(B)**, the apex as the primary auxin source is removed. Biosynthesis of SL is not promoted anymore, while repression of CK biosynthesis is released. Furthermore, the auxin level in the main stem is reduced and thus the sink capacity is increased, facilitating the establishment of an initial auxin export from the bud. After bud outgrowth, the emanating branch takes over the function of the lost apex as the primary auxin source and re-establishes apical dominance. Both described models, the second messenger model and the auxin transport canalization model, are not mutually exclusive, and the described pathways could contribute to bud outgrowth control simultaneously. After bud outgrowth, the angle of the branch is also under control. TILLER ANGLE CONTROL1 (TAC1) increases the tiller angle in monocots, while LAZY1 has the opposite function and reduces the tiller angle. Black lines and letters designate active pathways; light gray lines and letters indicate suppression or down-regulation of the respective pathway.

The architecture of mature plants is determined by the frequency of axillary meristem initiation, the control of bud outgrowth, as well as subsequent dynamics in branch growth. Variation of these parameters generates the high morphological diversity observed in different plant species and even between individuals within a given species. This variation is largely based on genetic predisposition. However, the architecture that is characteristic of a plant species may be modified in response to environmental conditions. An important parameter of modification is the activity of axillary buds. Axillary branching is normally suppressed or at least reduced by the shoot apex through a regulatory system that has been termed apical dominance (reviewed in Cline, 1997; Leyser, 2005). The basic principles that govern bud outgrowth control have been described several decades ago. Snow (1925) could show that maintenance of apical dominance needs a signal that moves downward from a dominant shoot apex and, in addition, another signal may be transported upward into the dormant bud to suppress outgrowth. Thimann and Skoog (1933) identified the plant hormone auxin as the downward signal. Auxin, mainly synthesized in expanding young leaves of the plant apex (Ljung et al., 2001), is transported basipetally in the stem. Removal of the apical auxin source by decapitation abolishes apical dominance, while application of auxin to the apex of these decapitated plants can restore apical dominance (Thimann and Skoog, 1933). However, the inhibitory effect of auxin is not direct. It was shown that external auxin application to axillary buds does not prevent their outgrowth and experiments with radiolabeled auxin revealed that apex-derived auxin does not enter the dormant bud. Additionally, auxin transport appears to be too slow to mediate a direct effect (Hall and Hillman, 1975; Morris, 1977; Everat-Bourbouloux and Bonnemain, 1980; Booker et al., 2003). As a consequence of these studies, a long distance second messenger was postulated. According to this model, such a second messenger relays the downward auxin signal upward into the dormant bud. There are two good candidates for this messenger: cytokinins and strigolactones. Cytokinin is produced in roots and the stem and transported acropetally in the xylem (Nordstrom et al., 2004). Manipulations of plant cytokinin content show clear effects on bud outgrowth control, e.g., application of cytokinin to axillary buds releases dormancy even in plants that have an intact apex (Sachs and Thimann, 1964). Thus, with respect to bud outgrowth control, cytokinins act antagonistically to auxin. Most likely, the readout of auxin-cytokinin crosstalk generates part of the signaling chain that controls dormancy. The question of how auxin influences cytokinin as a second messenger was addressed by Nordstrom et al. (2004), who found that auxin can dampen cytokinin biosynthesis (**Figure 2A**). Basipetally transported auxin from the plant apex decreases expression of the cytokinin biosynthesis gene *ISOPENTENYLTRANSFERASE* (*IPT*) in the stem (Tanaka et al., 2006). In addition, it was shown for pea stems that auxin induces the cytokinin oxidase gene *PsCKX2* (Shimizu-Sato et al., 2009). Cytokinin oxidases inactivate cytokinin and, thus, lower the pool of active cytokinin (Werner et al., 2001). As a consequence of decreased biosynthesis and increased degradation, the cytokinin content is lowered in the stem and bud dormancy is maintained. In contrast, the decrease of auxin in the stem after removal of the main auxin biosynthesis site by decapitation will lead to increased cytokinin biosynthesis (Bangerth, 1994; **Figure 2B**). In pea, the *PsIPT1* and *PsIPT2* genes are induced in the nodal stem near the axillary buds after decapitation. Consistently, increased cytokinin levels could be detected in excised nodal stems (Tanaka et al., 2006). Cytokinin may then be transported into the adjacent buds. Indeed, it was shown in pea that the zeatin riboside content increased in axillary buds after decapitation (Turnbull et al., 1997).

In addition to damages to the apex, other environmental impacts such as nutrient availability (e.g., nitrogen or phosphorus concentrations in the soil) or planting density-related shading, also profoundly change plant architecture (Casal et al., 1986; Lopez-Bucio et al., 2002; Yoneyama et al., 2013; de Jong et al., 2014). The developmental response of the plant shoot to nutrient supply most likely involves a long distance, graft-transmittable signal from the root. Root tips are a main biosynthesis site of cytokinin (Miyawaki et al., 2004; Nordstrom et al., 2004) and it is tempting to speculate that changes in the cytokinin export from the root to the shoot via the xylem stream provide the postulated long distance signal for root–shoot communication. However, Faiss et al. (1997) showed in grafting experiments that transgenic roots overproducing cytokinin could not induce bud outgrowth in wild type scions, which made cytokinin unlikely to be the elusive signal. Analyses of branching mutants in *Arabidopsis* (*more axillary branching -max*), pea (*ramosus* -*rms*), petunia (*decreased apical dominance*-*dad*), and rice (*dwarf* –*d*) finally led to the discovery of the shoot branching hormone strigolactone (SL; Gomez-Roldan et al., 2008; Umehara et al., 2008) which features the required characteristics of the sought-after long distance signal in branching control: it inhibits shoot branching (Gomez-Roldan et al., 2008; Umehara et al., 2008), it can be transmitted from wild type roots to mutant shoots via grafting and complements the branching phenotype (Beveridge et al., 1997; Sorefan et al., 2003; Booker et al., 2005; Beveridge, 2006). Acropetal SL transport was shown to occur in the xylem (Kohlen et al., 2011) and the biosynthesis is increased by auxin (Sorefan et al., 2003; **Figure 2A**).

After the discovery of SLs as branching hormones, much effort was put in unraveling their biosynthesis and signaling pathways. SL biosynthesis starts from carotenoid precursors via the action of the all-*trans*/9-*cis*-β-carotene isomerase D27 in rice and AtD27 in *Arabidopsis* (Lin et al., 2009; Waters et al., 2012). Subsequent processing is carried out by the carotenoid cleavage monooxygenases CCD7 and CCD8. These enzymes are known in several species and named MAX3 and MAX4 in *Arabidopsis* (Sorefan et al., 2003; Booker et al., 2004), RMS5 and RMS1 in pea (Sorefan et al., 2003; Johnson et al., 2006), D17 and D10 in rice (Ishikawa et al., 2005; Arite et al., 2007; Alder et al., 2012), and DAD1 in petunia (Snowden et al., 2005).

Successful complementation of *Arabidopsis max* mutants with putative *MAX* orthologs from willow and poplar and response of willow buds to the SL analog GR24 indicate that SLs are also synthesized and perceived in woody plants such as trees (Ward et al., 2013; Czarnecki et al., 2014).

In addition to CCD7 and CCD8, the cytochrome P450 monooxygenase MAX1 is involved in downstream SL biosynthesis in *Arabidopsis* (Stirnberg et al., 2002; Booker et al., 2005; **Table 1**). An overview about all aforementioned SL biosynthesis genes can be found in **Table 1**.

**Table 1:** Genes involved in strigolactone biosynthesis and signaling.

*Arabidopsis*	Pea	Petunia	Rice	Function	Regulation
*MAX1*			*SLB1, SLB2*	P450 cytochrome	

*MAX2*	*RMS4*	*PhMAX2A, PhMAX2B*	*D3*	F box protein	

*MAX3*	*RMS5*		*D17*/*HTD1*	CCD7 carotenoid cleavage dioxygenase	Auxin maintains transcript levels (pea)

*MAX4*	*RMS1*	*DAD1*	*D10*	CCD8 carotenoid cleavage dioxygenase	Auxin maintains transcript levels (pea), decapitation decreases transcript levels, deficiency in SL biosynthesis, or signaling increases transcription (effect depends on RMS2)

	*RMS2*			Generation of mobile shoot to root signal	

*AtD14*		*DAD2*	*D14*	α/β-fold hydrolase; perception of SL	

*SMXL6*; *SMXL7*; *SMXL8*; *SMAX1*			*D53*	HSP101/chaperonin-like	

*The information in the table was taken from Beveridge et al. (1997, 2000), Foo et al. (2005), Johnson et al. (2006), Braun et al. (2012), Drummond et al. (2012), and Cardoso et al. (2014).*

Strigolactone biosynthesis generally occurs in roots and in the shoot (Auldridge et al., 2006; Umehara et al., 2008; Mashiguchi et al., 2009). Grafting studies revealed that wild type rootstocks can suppress the phenotypes of *max1, max31*, and *max4* biosynthesis mutant scions (Sorefan et al., 2003), indicating that SL or a SL precursor can travel from root to shoot (Turnbull et al., 2002; Beveridge, 2006). The product of the CCD8 reaction, carlactone, has been discussed as a possible mobile SL precursor (Seto and Yamaguchi, 2014). This hypothesis is based on the observation that a *max1* rootstock can complement the branching phenotype of a *max4* scion (Booker et al., 2005). Recently, Abe et al. (2014) analyzed the MAX1 reaction in SL biosynthesis in detail. They demonstrated that carlactone is converted to carlactonic acid by the action of MAX1 in *Arabidopsis*. However, subsequent reactions to generate bioactive SLs remain to be elucidated. The same authors reported hints for the interaction of a carlactonic acid methyl ester with the putative SL receptor AtD14 (see SL signaling discussion below; Abe et al., 2014). Therefore, we are close to fully understanding the biosynthesis pathway of at least one bioactive SL. However, there are multiple other bioactive variants of SLs. The detailed reactions leading to this diversity as well as possible alternative biosynthesis pathways remain to be discovered.

Grafting experiments also revealed a class of SL response mutants that could not be complemented by wild type rootstocks (Beveridge et al., 1996; Stirnberg et al., 2007), indicating a role in SL perception and signaling rather than biosynthesis. An example is the *Arabidopsis max2* mutant (Stirnberg et al., 2007) that encodes an F-box protein involved in SL signaling. Its counterparts in rice and pea were described previously (Ishikawa et al., 2005; Johnson et al., 2006; **Table 1**). Within 6 years after the first description of SLs as branching hormones, further key components of SL signaling have been identified and a tentative scaffold of the signal transduction pathway has been assembled (Bennett and Leyser, 2014; Waldie et al., 2014). The α/β hydrolase D14 is most likely a receptor for SL. *d14* mutants in *Arabidopsis*, petunia and rice are insensitive to treatment with the SL analog GR24 and show an increased branching phenotype. Also, D14 exhibits high and specific affinity to GR24 (Kagiyama et al., 2013). In the presence of GR24, the petunia D14 ortholog DAD2 interacts with PhMAX2 (Hamiaux et al., 2012). This indicates that in analogy to other plant hormone signaling pathways, D14 may interact with the F-box protein MAX2 upon SL binding, leading to ubiquitination-mediated degradation of a SL signaling repressor (Bennett and Leyser, 2014). Since *max2* mutants show pleiotropic effects, it is likely that MAX2 interacts with several pathways and may mediate degradation of different target proteins. Indeed, three different candidate repressors for the strigoalactone signaling pathway have been identified: DELLA proteins (Nakamura et al., 2013), BES1 (Wang et al., 2013), and D53 in rice (Jiang et al., 2013; Zhou et al., 2013). While interaction of MAX2 with DELLA proteins and BES1 may point to cross talk with the gibberellic acid and brassinosteroid pathway, respectively, D53 emerges as the genuine SL pathway repressor (reviewed in Bennett and Leyser, 2014). Dominant gain of function mutations in D53 prevent SL-mediated degradation of the protein and shut off SL signaling. Moreover, rice D53 interacts with D3, which is the rice ortholog of MAX2, and *d3* mutants are suppressed by knockdowns of D53 (Jiang et al., 2013; Zhou et al., 2013). A possible *Arabidopsis* ortholog of D53 is SUPPRESSOR OF MORE AXILLARY GROWTH2 LIKE 7 (SMXL7; Stanga et al., 2013; Bennett and Leyser, 2014; **Table 1**). Interestingly, the basic principle of SL signaling is similar to auxin, jasmonic acid, and gibberellic acid signaling (reviewed in McSteen and Zhao, 2008). Briefly, binding of the hormone to a receptor activates an F-box protein-containing SCF E3 ligase complex, which mediates ubiquitination and subsequent degradation of a transcriptional repressor. Ultimately, this leads to changes in transcription of a specific set of genes (Hagen and Guilfoyle, 2002; Hartweck, 2008; Memelink, 2009).

Summarized, cytokinin and SL were shown to regulate bud outgrowth, but the mechanism of bud dormancy control and the reciprocal effect of these plant hormones had to be integrated into a model. Prusinkiewicz et al. (2009) and Balla et al. (2011) suggested combining the second messenger model with a model introduced by Li and Bangerth (1999). Their model of “autocorrelative inhibition” is based on the auxin canalization hypothesis by Sachs (1981) and discusses a competition of buds for establishment of a polar auxin transport stream (PATS). The auxin canalization hypothesis (reviewed in Domagalska and Leyser, 2011) suggests a feed forward mechanism to explain the establishment of polar auxin transport routes that induce the development of vascular tissues. Starting from an auxin source that provides a high auxin concentration, competent cells will transport auxin away from the source and establish an auxin gradient across the tissue. From this initial auxin flow, continuous transport will build up, keeping a high auxin concentration in the transport competent cells and subsequently increasing the expression and polarization of auxin carriers in these cells. As a consequence, auxin transport will further strengthen in a feed forward loop, which sustains and enhances transport competence in files of specific cells. Along these transport routes, vascular tissue will differentiate.

Research on the PIN auxin eﬄux carrier proteins provided experimental support for the canalization model. Biosynthesis and plasma membrane localization of PIN proteins are elevated by auxin (Paciorek et al., 2005) and the expression of PIN proteins precedes vascular development (Sauer et al., 2006; Scarpella et al., 2006; Wenzel et al., 2007). This model can be adapted for a hypothesis on the mechanisms that control apical dominance. As an initial auxin gradient is a prerequisite for the development of a PATS, only buds that achieve to build up an auxin gradient between the bud as an auxin source and the stem as a common auxin sink have the ability to establish a PATS and grow out. Usually, the actively growing apex is the main auxin source (**Figure 2A**). According to the auxin canalization model, apical dominance is therefore exerted by the apex through saturation of the auxin transport capacity of the stem, acting as an auxin sink. As a consequence, axillary buds are prevented from successfully establishing an initial auxin flux. Hence, they remain dormant.

After removal of the dominant apex, e.g., by decapitation, the auxin level in the stem decreases. The resulting increase in the sink capacity of the stem facilitates an initial auxin flux from dormant buds into the stem, finally releasing the dormancy of buds in the neighborhood of the formerly dominant shoot tip (**Figure 2B**). As soon as one or few buds grow out, the growing branches re-establish apical dominance by exporting auxin to the main stem. The sink capacity of the stem is consequently reduced back to normal levels, preventing further dormant buds from growing out.

Both models, the second messenger model and the model of autocorrelative inhibition/auxin canalization, are complementary. Cytokinin and SL, respectively, influence sink strength of the stem through changes in auxin biosynthesis and modification of PATS. As a consequence of decapitation, the inhibitory effect of auxin on cytokinin biosynthesis is dampened and increased cytokinin levels might enhance local auxin biosynthesis in the bud, increasing its auxin source strength. At the same time, the sink capacity of the stem may be enhanced by a cytokinin-mediated induction of the PATS in the stem by increased synthesis and polarization of PIN auxin eﬄux carriers. Indeed, such increased expression and polarization was shown for PsPIN1 in axillary buds after external cytokinin application (Kalousek et al., 2010). Furthermore, Marhavy et al. (2014) postulated a role for cytokinin in modulating AtPIN1 abundance and polarization during lateral root organogenesis.

In contrast to cytokinin, SL appears to decrease the amount of the PIN auxin eﬄux carrier at the membrane and, thus, lower auxin transport capacity in the stem. This was observed in stems of *Arabidopsis* SL-pathway mutants, which showed increased AtPIN1 levels as well as an increased auxin transport (Bennett et al., 2006; Prusinkiewicz et al., 2009). According to the auxin transport canalization model, SLs will, therefore, aggravate the establishment of auxin export from axillary buds, leading to increased apical dominance (Prusinkiewicz et al., 2009). Decapitation triggers down-regulation of SL biosynthesis gene *CCD8* transcript levels (Foo et al., 2005), most likely resulting in reduced SL biosynthesis. Such a reduction of SL levels would cause a release from their antagonistic effect on PIN polarization. As a result, an increased auxin flux to the root would occur and, thus, further increase the sink capacity of the stem. Summarized, high cytokinin and low SL levels may increase source strength of the bud and increase sink capacity of the stem, and, thus, facilitate the successful establishment of an auxin gradient. This gradient would allow an initial auxin flow from the bud to the stem and the establishment of vascular tissue as a prerequisite for bud outgrowth (**Figures 2A,B**). Already Sorokin and Thimann (1964) observed that a vascular connection between axillary buds and the main stem coincides or precedes bud outgrowth.

A drawback of the hypotheses on apical dominance control by auxin is the discrepancy between auxin transport velocity and bud outgrowth kinetics after decapitation. In decapitated pea plants, buds start to grow out before auxin concentrations in the associated nodal stem are diminished due to removal of the apical auxin source (Morris et al., 2005). Thus, an alternative primary messenger is discussed. Mason et al. (2014) reported that after decapitation, sucrose concentrations in axillary buds increased. Moreover, buds could be released from dormancy by sucrose treatment and inhibition of sucrose transport by girdling prevented outgrowth of buds. Importantly, the measured speed of sucrose transport is sufficient to relay the signal from the shoot apex to a dormant axillary bud in time before first signs of bud outgrowth occur. Mason et al. (2014) therefore suggest that the primary signal after decapitation is sucrose and that auxin controls the number of buds that will grow out. The observation that the branching suppressor *BRANCHED1* (*BRC1*) is down-regulated after sucrose treatment provides further arguments for this “nutritive hypothesis,” whose general concept was postulated earlier (reviewed in Phillips, 1975).

## BRANCHED1 is a Key Factor in Bud Outgrowth Control

BRANCHED1 (BRC1) is a TB1 CYCLOIDEA PCF (TCP) type transcription factor (Aguilar-Martinez et al., 2007; Finlayson, 2007). Proteins of this group are either assigned to class I which contains PCF-like proteins or class II which consists of CYCLOIDEA/TB1-like proteins. It has been suggested that class I TCP factors increase cell division rates, while class II TCP factors inhibit cell cycle progression (Martin-Trillo and Cubas, 2010). The protein group takes its name from the TCP domain which is a highly conserved 59 amino acid basic helix-loop-helix structure that mediates DNA binding, protein–protein interaction, and nuclear targeting. Class II TCP transcription factors that regulate axillary meristem activity have been identified in several plant species (Doebley et al., 1997; Takeda et al., 2003; Kebrom et al., 2006, 2010; Aguilar-Martinez et al., 2007; Finlayson, 2007; Minakuchi et al., 2010; Martin-Trillo et al., 2011; Braun et al., 2012). Even slight expression changes of these factors profoundly modify plant architecture, as it was described for TB1 levels in maize compared to its anticipated ancestor teosinte (Doebley et al., 1997). Orthologs of maize *TB1* were identified in other monocots like rice (*FINE CULM1*/*OsTB1*) and sorghum (*SbTB1*; Takeda et al., 2003; Kebrom et al., 2006). Aguilar-Martinez et al. (2007) and Finlayson (2007) described the *TB1* orthologs *BRANCHED1* (*BRC1*= *TCP18*) and *BRANCHED2* (*BRC2*= *TCP12*) in the dicot species *Arabidopsis*. The fact that *Arabidopsis* contains two *BRC* paralogs is due to duplications of the *Arabidopsis* genome (Franzke et al., 2011; Vanneste et al., 2014). With respect to axillary branching, *BRC1* seems to be the major regulator, while *BRC2* shows a comparably low expression and *brc2* knockout lines exhibit weaker phenotypes compared to *brc1* plants (Aguilar-Martinez et al., 2007; Finlayson, 2007). *BRANCHED1* genes were also identified in tomato (*SLBRC1a* and *b*; Martin-Trillo et al., 2011) and pea (*PsBRC1*; Braun et al., 2012). In accordance with *BRC1* being a suppressor of branching, *brc1* knockout mutants have more rosette branches. While in wild type *Arabidopsis* plants less than 40% of buds grow out, almost 100% of rosette buds elongate and form a branch in *brc1* plants (Aguilar-Martinez et al., 2007). In addition, leaf axils of cotyledons in *brc1* plants sometimes develop axillary meristems that form buds and grow out. In contrast, leaf axils of cotyledons never develop axillary buds in wild type plants. This indicates that *BRC1* not only controls bud outgrowth, but also regulates axillary meristem initiation. Leaf axils of cauline branches (shoots of the inflorescence) are not affected in *brc1* knockout lines. Thus, BRC1 specifically controls axillary meristem initiation and bud outgrowth in rosette leaf axils.

The *BRC1* expression pattern correlates well with the anticipated role of BRC1 as a repressor of cell division and bud outgrowth. As revealed by Northern Blot, qPCR and *in situ* hybridization experiments, *BRC1* expression is high in dormant rosette leaf buds and low in elongating, i.e., growing buds (Aguilar-Martinez et al., 2007; Finlayson, 2007). In addition, a gradient of *BRC1* expression exists along the apical to basal axis in rosette leaf buds of *Arabidopsis* grown under long day conditions. Young buds near the shoot apex exhibit low *BRC1* expression levels and older buds at the base of the rosette contain high amounts of *BRC1* transcript (Finlayson, 2007). This coincides with the basipetal wave of axillary bud initiation and outgrowth in *Arabidopsis* after onset of flowering, i.e., buds with lower basal *BRC1* levels grow out earlier (Hempel and Feldman, 1994). In other investigated tissues than buds, *BRC1* transcript levels are very low or non-detectable (Aguilar-Martinez et al., 2007; Finlayson, 2007), emphasizing its specific role in the regulation of bud outgrowth.

In order to investigate its subcellular localization, Aguilar-Martinez et al. (2007) expressed BRC1 as GFP fusion under the control of the constitutively and ubiquitously active *35S* promoter and showed that BRC1 is localized in the nucleus. With these *p35S:GFP:BRC1* plants, they observed a severely stunted growth phenotype (Aguilar-Martinez et al., 2007), which is probably the result of misexpression of *BRC1* at the shoot apex, further underlining its role as a growth repressor. Taken together, these observations indicate that in dicots, BRC1 acts as a transcriptional regulator that inhibits cell division in axillary buds. It was suggested that a final target of the signaling chain that involves TB1/BRC1 may be factors like PCNA that regulate the cell cycle (Müller and Leyser, 2011).

Expression of maize *TB1* in wheat from its native maize promoter (Lewis et al., 2008) or *OsTB1* in rice using the strong and constitutive rice actin promoter (Takeda et al., 2003) did not decrease plant growth but specifically affected outgrowth of axillary buds. Investigations by Guo et al. (2013) indicate that in monocots, TB1 may have a different mode of action than in dicots and may explain why rice OsTB1 overproducers do not show growth depression. Guo et al. (2013) identified the MADS box factor OsMADS57 that functions to increase tillering. Tillers are axillary branches that originate from the shoot base of monocots (**Figure 1B**). OsMADS57 is a transcriptional repressor that down-regulates expression of the SL receptor *DWARF14*. TB1/BRC1 in turn directly interacts with the OsMADS57 protein and, thereby, inactivates OsMADS57. As a consequence DWARF14 expression is de-repressed and SL perception is increased. Thus, in monocots TB1/BRC1 may not repress progression of the cell cycle, but control outgrowth of axillary buds by enhancement of SL signaling.

## BRANCHED1 is a Central Integrator of Endogenous and Environmental Factors that Modulate Branching

### Endogenous Factors/Hormonal Regulation

In order to investigate a possible influence of auxin on *BRC1*, Aguilar-Martinez et al. (2007) and Finlayson (2007) analyzed *BRC1* expression in rosette buds of *35S:YUCCA* plants that exhibit increased apical dominance due to auxin overproduction. Aguilar-Martinez et al. (2007) reported no effect of increased auxin levels on *BRC1* expression in these plants. However, Finlayson (2007) determined *BRC1* expression in upper and lower buds separately and found a significant increase in upper buds of *35S:YUCCA* plants compared to wild type plants. Therefore, auxin seems at least partially to play a role in influencing *BRC1* expression. Direct application of cytokinin on buds reduced *BRC1* transcript levels in pea (Braun et al., 2012; Dun et al., 2012). Also in rice, cytokinin application decreased *FINE CULM1* (*FC1*) expression (Minakuchi et al., 2010). In accordance with these observations *Arabidopsis altered meristem program1* (*amp1*) mutants, which show increased cytokinin levels, exhibit slightly decreased *BRC1* expression and more branches than wild type plants (Aguilar-Martinez et al., 2007).

The strongest effect on *BRC1* transcript levels was observed in *max1, max3, max4* SL biosynthesis mutants. The down-regulation of *BRC1* expression in *Arabidopsis max* mutants indicates that SLs regulate *BRC1* transcriptionally (Aguilar-Martinez et al., 2007). Data in favor of the hypothesis that BRC1 acts downstream of SLs has also been obtained from investigations in pea. Studies showed that *PsBRC1* transcript levels are upregulated by SL application and down-regulated in SL synthesis and signaling mutants (Braun et al., 2012; Dun et al., 2013). In turn, rice *fc1* knockout mutants did not respond to SL (Minakuchi et al., 2010) and also in *Arabidopsis*, GR24 treatment did not repress the increased branching phenotype of the *Atbrc1* mutant (Brewer et al., 2009). In contrast, overexpression of FC1 could not suppress the branchiness of SL mutants (Minakuchi et al., 2010) and *FC1* expression remains high in buds of SL mutants (Arite et al., 2007). These results appear to be contradicting and may be explained by other branching pathways in which SLs are involved (e.g., modulation of auxin transport, see auxin canalization model) as well as the fact that BRC1 is not solely regulated by SLs. BRC1 was proposed to be a central integrator of different branching pathways (Aguilar-Martinez et al., 2007).

Summarized, there appears to be an effect of the three main branching hormones auxin, cytokinin and SL on *BRC1* (**Figure 2A** and **Table 2**), and further pathways seem to play a role (Rameau et al., 2015).

**Table 2:** Regulation of *BRC1*/*TB1* expression.

Hormone	*Arabidopsis AtBRC1*	Pea *PsBRC1*	Rice *FINE CULM*	Feedback regulation
Auxin	Down-regulation in buds of younger rosette axils of *35S:YUCCA* (auxin overproducer)			
Cytokinin	Down-regulation in *amp1* mutants (higher cytokinin levels)	Down-regulation, effect is independent of SL	Down-regulation	
Strigolactone/ GR24	Down-regulation in SL deficiency or signaling mutants (*max*)	Upregulation, low transcript levels in *rms1* and *rms4* SL mutants	No effect	SL levels are higher in *Psbrc1* mutants (pea)

*The information in the table was taken from Aguilar-Martinez et al. (2007), Finlayson (2007), Minakuchi et al. (2010), Braun et al. (2012), and Dun et al. (2012).*

### Exogenous Factors/Shading

The signaling chains of auxin, cytokinin, and SL are modulated by environmental factors like shading or the plant nutritional status. The level of shading by neighboring plants is a measure for population density and, thus, an indicator of competition for light. Red light is absorbed by plants, while far red light is largely transmitted through the leaf canopy. As a consequence, shading by other plants reduces the ratio of red light to far red light (R/FR). Plants quantify this ratio through the phytochrome system and react with the shade avoidance syndrome that enables plants to outgrow the competitors for light (Casal, 2013; Pierik and de Wit, 2014). By suppression of branching, more resources are allocated to the main shoot and, consequently, the growth rate of the shoot increases and the plants grow taller in a shorter period of time.

The photoreceptor phytochrome can adopt two different conformations: Pr and Pfr. Upon absorption of red light, Pr (inactive) is converted to Pfr (active) which shuttles to the nucleus and controls gene expression through interaction with PHYTOCHROME INTERACTING FACTOR (PIF) or PIF3-like (PIL; Leivar and Monte, 2014) Within the family of five phytochromes in *Arabidopsis*, mainly phyB was shown to control red light responses of plant architecture (Finlayson et al., 2010; Reddy and Finlayson, 2014). In sorghum, low R/FR ratios or knockout of phyB prevented bud outgrowth, which was correlated with high *TB1* transcript levels in axillary buds (Kebrom et al., 2006). It was hypothesized that phyB suppresses *TB1*/*BRC1* and that the high FR proportion of light in a dense plantation will convert active phyB Pfr to inactive phyB Pr and thus, suppress bud outgrowth via increased *TB1/BRC1* expression. Similarly, knockout of *phyB* increases *TB1/BRC1* levels and therefore, reduces bud outgrowth. The observation that the *Arabidopsis* knockout mutant *brc1-2* does not show branching suppression under low R/FR conditions supports the hypothesis that *TB1/BRC1* plays a central role in branching suppression during shade avoidance (Gonzalez-Grandio et al., 2013). A putative downstream target of TB1 during the shade avoidance response in maize is the HOMEODOMAIN-LEUCINE ZIPPER (HD-ZIP) protein GRASSY TILLERS1 (GT1; Whipple et al., 2011). *GT1* is expressed in leaf primordia of axillary buds and in provascular tissue below the axillary bud. Interestingly, signals of GFP-tagged GT1 were observed in cells of the axillary meristem, indicating non-cell-autonomous activity of GT1. Comparable to *tb1* loss-of-function mutants, *gt1-1* knockout mutants exhibit an increased branching phenotype. The significantly reduced *GT1* expression in *tb1* mutants indicates that TB1 and GT1 act in the same pathway. Since *TB1* expression is not changed in *gt1* mutants, it is likely that TB1 acts upstream of *GT1* and regulates its expression (**Figure 2A**). Light conditions with a low R/FR ratio induce the expression of GT1, indicating that suppressed branching during the shade avoidance syndrome is due to TB1-mediated upregulation of *GT1* expression.

Plants that suffer from suboptimal nutrient supply also exhibit decreased branching comparable to plants that compete for light. However, in contrast to the shade avoidance syndrome, during nutrient deprivation resources are not allocated to the shoot but instead to the root to facilitate enhanced nutrient uptake from the soil. Nutrient-induced changes in shoot/root ratio and root development are most obvious with plants grown under phosphate deficiency (Forde and Lorenzo, 2001; Lopez-Bucio et al., 2002). Branching in these plants is suppressed and many lateral roots develop near the soil surface, which was termed “topsoil foraging” (Peret et al., 2014). These changes in root morphology increase phosphate uptake from soil layers that are enriched in phosphate (Peret et al., 2011; Niu et al., 2013; Hunter et al., 2014).

Kohlen et al. (2011) quantified the number of shoot branches of *Arabidopsis* wild type and SL biosynthesis (*max1*, *max4*) and signaling (*max2*) mutants under phosphate sufficient and phosphate deficient conditions. Branching of wild type plants was significantly reduced under phosphate deficiency while none of the *max* mutants responded to low phosphate. The observed difference in branching suppression correlated with the SL content of the xylem sap. The strigolactone orobanchol could be detected in root exudates and xylem sap of wild type *Arabidopsis* and showed an increase in concentration when *Arabidopsis* was grown on phosphate deficient substrate. In contrast, the root exudate of the SL biosynthesis mutants *max1* and *max4* that were unresponsive to phosphate deficiency did not show an increase in SL content under phosphate-limiting conditions (Kohlen et al., 2011). Phosphate starvation increased SL synthesis also in tomato (Lopez-Raez et al., 2008), sorghum (Yoneyama et al., 2007), and rice (Umehara et al., 2010). Umehara et al. (2010) showed that the rice SL biosynthesis genes *D17* (*MAX3* in *Arabidopsis*) and *D10* (*MAX4* in *Arabidopsis*) are induced by low phosphate conditions.

Similarly to phosphate, also the nitrogen supply influences plant architecture. Low nitrogen suppresses branching and changes the root/shoot ratio toward higher root biomass proportions (Forde and Lorenzo, 2001; Euring et al., 2012). Quantification of SLs in roots and root exudates of sorghum and pea plants grown under low nitrogen conditions showed that nitrogen deficiency increased SL levels in these plants (Yoneyama et al., 2007; Foo et al., 2013), which points to SL-mediated suppression of bud outgrowth under nitrogen limitation. Vice versa, optimal nitrogen supply decreases SL production, which may lead to increased branching (Yoneyama et al., 2013).

A hallmark of SL activity is the decrease of auxin transport in the main stem via decrease of PIN1 levels at the plasma membrane, as mentioned earlier (Prusinkiewicz et al., 2009). However, auxin transport capacity in the main stem of *Arabidopsis* is not diminished by low nitrogen (de Jong et al., 2014), but, instead, auxin supply to the PATS from the main shoot apex is higher in nitrogen starved plants. The increased auxin biosynthesis at the apex makes it a stronger auxin source, reducing the sink strength of the PATS relative to the axillary buds. According to the canalization hypothesis (Sachs, 1981), this weak sink strength will prevent establishment of a PATS from axillary buds and, thus, consolidate bud dormancy. Analyses of *Arabidopsis* mutants showed that intact auxin signaling and SL biosynthesis are both required for increased supply of auxin from the shoot apex leading to suppression of branching under nitrogen starvation (de Jong et al., 2014).

In conclusion, phosphate and nitrogen supply of the plant clearly affect plant architecture and SLs are involved in the plant responses to nutrient supply. However, the mechanism leading to a change in branching may vary in different plant species.

The nutrient- or shading-induced changes in plant architecture exemplify that plants can adapt their branching patterns to the prevailing environmental conditions. This demonstrates that plant architecture closely correlates with plant growth and survival. Likewise, crop plant performance is determined by branching characteristics and it is not surprising that during domestication of crop plants, certain architectural traits were a major target for selection of improved cultivars. Especially monocot crops like rice, sorghum, maize, and wheat are of great importance for world nutrition. The architectural diversity of monocot plants allowed the selection of specific architectural traits from a broad natural gene pool during domestication.

## Branching Relevant Genes Selected during Domestication and Plant Breeding

Monocot crop plants belong to the grasses which have been assigned to two major clades, consisting of subfamilies (Barker et al., 2001). Cereals of the first clade, which are important for world nutrition, belong to the subfamily Ehrhartoideae (including rice) and the Pooideae (including oat, wheat, barley, rye). Within the separate, second major clade is the subfamily of the Panicoideae with maize, sorghum, and millets. Grasses exhibit two types of vegetative branching patterns (Doust, 2007), depending on the position of branch development with respect to the plant main axis. Tillers are typical for many grasses and determine their characteristic growth habit (**Figure 1B**). Tillers are branches that originate from nodes near the plant basis. These branches reach a similar height like the main stem and have the capacity to form adventitious roots. Axillary branches that initiate at upper positions of the culm (the main stem of grasses) are similar to branches of dicot plants. Grasses of the two major phylogenetic clades can be classified according to these branching patterns. Plants of the Ehrhartoideae and the Pooideae develop many tillers and no axillary branches while members of the Panicoideae produce tillers and, in addition, initiate axillary meristems that can grow into axillary branches (Doust, 2007).

The architectural traits selected during the domestication of crop plants include the extent of vegetative shoot and inflorescence branching, branch angle, as well as internode elongation. Inflorescence branching and genes involved in stem elongation like the DELLA genes (Peng et al., 1999; Sasaki et al., 2002) have been covered in recent reviews (Fernandez et al., 2009; Teo et al., 2014; Zhang and Yuan, 2014). Here, we will therefore focus on vegetative branching and branch angle.

Changes in vegetative branching phenotypes during plant domestication are most evident in monocot crop plants and the molecular bases of these changes have been thoroughly studied. During domestication of panicoid grasses, plant lines have been selected that show a decrease in both tillering and axillary branching. Modern cultivars of domesticated maize plants develop ideally only one female inflorescence (ear) and a high proportion of fixed carbon is allocated to the developing ear. Only the main stem terminates in a single male inflorescence (tassel). In contrast, wild forms of *Zea mays* subsp. *mays* (*Zea mays* subsp. *parviglumis* and *Zea mays* subsp. *mexicana*, collectively named teosinte) develop many axillary branches at the main stem which produce female inflorescences from secondary axillary meristems. Each branch terminates in a male inflorescence. Doebley et al. (1997) discovered that one of the quantitative trait loci that determine maize architectural changes during domestication carries the *TEOSINTE BRANCHED1* (*TB1*) gene. Small changes in expression strength of *TB1* seem to be sufficient to cause the significant differences in branching patterns between teosinte and maize (Doebley et al., 1997). Maize was domesticated in Mesoamerica (Holst et al., 2007; Piperno et al., 2007; Pohl et al., 2007), while the other monocot crops belonging to the *Panicoideae*, pearl millet and sorghum, were selected in Sub-Saharan Africa (Remigereau et al., 2011). Interestingly, comparative QTL mapping revealed that also in pearl millet, *TB1* was the molecular target of domestication (Remigereau et al., 2011). Polymorphism analyses comparing cultivated pearl millet with the wild form *Pennisetum glaucum* showed that the nucleotide diversity of the *TB1* gene dramatically dropped in a region upstream of the transcription start site. This analysis indicates that nucleotide changes important for the reduced branching of pearl millet occurred within the promoter region of the *TB1* gene (Remigereau et al., 2011). Such decreases of polymorphism restricted to single genes are characteristic of domestication events in contrast to evolutionary bottle necks that result in a reduction of polymorphism on the whole genome scale. Summarized, the studies in maize and pearl millet indicate that changes in the promoter activity and expression level of the domestication target gene *TB1* may be causal for the reduced branching of some monocot crops.

To combine the knowledge on economic aspects of monocot crop architecture and to define targets of monocot crop breeding, an architectural ideotype that exhibits the ideal plant architecture (IPA) has been described (Lu et al., 2013). With respect to rice, this ideotype is characterized by low tiller number, high tiller productivity and a thick and strong culm (Jiao et al., 2010; Lu et al., 2013). Jiao et al. (2010) and Miura et al. (2010) both analyzed rice varieties that show IPA characteristics. Map based cloning attempts to isolate the quantitative trait loci that determine IPA resulted in the isolation of *IPA1*/*OsSPL14*, which is expressed at the shoot tip and in developing branches. This gene is negatively regulated by the microRNA *OsmiR156*. The low tillering *Oryza japonica* lines ST-12 and Shaoniejing that were independently analyzed by Jiao et al. (2010) and Miura et al. (2010), respectively, carry a mutation in the *miR156* complementary site. Thus, in both lines, SPL14 mRNA is resistant to *miR156*-mediated degradation and accumulates to a higher RNA level than in the rice cultivars Nipponbare and Taichung Native 1 which were used as reference lines in map based cloning.

*IPA1/OsSPL14* encodes the transcription factor SQUAMOSA PROMOTER BINDING PROTEIN-LIKE 14. A DNA motif that is bound by IPA1/OsSPL14 was found in the *OsTB1* promoter (Lu et al., 2013). The fact that a transgenic rice line that produces a *miR156* resistant *IPA1/SPL14* mRNA exhibits higher *OsTB1* transcript levels indicates that IPA1/SPL14 positively regulates *OsTB1* expression. As described above, TB1 is an important target during domestication and increased expression of *OsTB1* leads to suppression of bud outgrowth, which most likely causes the observed low tillering phenotype of the analyzed rice lines with characteristics of IPA. However, low tillering is not the only characteristic of IPA. The *O. japonica* lines ST-12 and Shaoniejing also exhibit taller and stronger culms. This observation points to a pleiotropic action of IPA1/SPL14. In addition to bud outgrowth suppression caused by higher expression of *OsTB1*, increased plant height and higher grain number per panicle may be mediated by induction of *DENSE AND ERECT PANICLE1* (*DEP1*; Huang et al., 2009) through IPA1/SPL14 (Lu et al., 2013). Besides from cultivars with an altered *miR156* – *IPA1/SPL14* pathway which were selected by classical breeding during crop domestication, a biotechnological approach, in which *miR156* was overexpressed in switchgrass, was successful. The overexpressing lines exhibited increased tillering and also the biomass quantity and quality were improved, which is beneficial for the use of switchgrass as a resource of bioenergy (Fu et al., 2012).

Another example for tillering-relevant genes are STRIGOLACTONE BIOSYNTHESIS 1 and 2 (*SLB1* and *SLB2*). Cardoso et al. (2014) identified these closely related genes by QTL mapping in rice. They are present in the low-tillering cultivar Azucena (*Japonica* subspecies), while they are absent from the high-tillering cultivar Bala (*Indica* subspecies) due to a genomic rearrangement. Both genes show high orthology to the *Arabidopsis* SL biosynthesis gene *MAX1* and are functional in *Arabidopsis*, since they can rescue the *max1* mutant phenotype (Cardoso et al., 2014). More recently, they were shown to catalyze the oxidation and subsequent hydroxylation of carlactone to yield the SL orobanchol (Zhang et al., 2014). Consistently, the cultivar Bala exudes low SL levels from roots (Cardoso et al., 2014). A generally reduced SL production would explain the high tillering phenotype and indicate that SLs are also important regulators of the architecture of crop plants, besides from the factors discussed above.

The initial reason for the QTL mapping, however, was not plant architecture. SLs are exuded by roots into the rhizosphere, where they promote arbuscular mycorrhiza, especially under phosphate starvation conditions. Root parasitic plants, such as *Striga*, appear to exploit this mechanism and use SLs as germination cues (reviewed in Bouwmeester et al., 2007). SLs therefore induce germination of *Striga* seeds, which is in line with the finding that the rice cultivar Azucena, exhibiting high SL exudation, is more susceptible to *Striga* infection (Cardoso et al., 2014). Therefore, SLs also play an important role in plant resistance in addition to their function in the control of rice tillering. Thus, they are a potent target for breeding efforts for improving agronomical traits in crop plants. Furthermore, a recent publication indicates that SL genes may also be a quantitative trait in trees used on short rotation plantations. The willow ortholog of *MAX4* co-localizes with a QTL for shoot resprouting after coppicing (Salmon et al., 2014). Thus, manipulation of the SL pathway for improvement of crop plants may specifically be useful for fast growing trees like willow and poplar which are cultivated on short rotation coppices. These trees are grown for 3–5 years and, after harvesting, the plants are allowed to resprout from the stool to start the next rotation.

In addition to the degree of tillering, the angle between tiller and culm determines the suitability of rice varieties for rice farming (Wang and Li, 2005). Tillers of the wild rice *Oryza rufipogon* grow in a horizontal orientation during the vegetative phase. This horizontal growth habit suppresses competing weeds, but the horizontal tillers have high space requirements and are not suitable for cultivation of rice in dense stands. Thus, rice varieties with a more compact growth due to a smaller tiller angle were selected during domestication.

Yu et al. (2007) isolated *TILLER ANGLE CONTROL1* (*TAC1)* by map-based cloning in an attempt to characterize a quantitative trait locus that decreases the tiller angle in rice. They used a mapping population obtained from a rice variety with almost zero tiller angle (straight tillers, compact growth) and a line with spread out tillers. The rice variety with compact growth carries a mutation in the 3′UTR of *TAC1* which leads to aberrant splicing. The resulting mRNA contains a mutated 3′UTR that leads to decreased stability. Yu et al. (2007) could show that high levels of *TAC1* mRNA correspond to a large tiller angle and low expression levels to a smaller tiller angle, respectively. Analysis of 152 rice accessions (wild type, *O. japonica* and *O. indica* cultivars) revealed that all lines with low tiller angle carry the identical *tac1* mutation that leads to aberrant splicing of the *tac1* transcript (Yu et al., 2007).

*TAC1* shows sequence similarity to *LAZY1*, a gene that is also involved in tiller angle determination. In contrast to *tac1*, a loss of function in *lazy1* results in wider tiller angles. This effect on tiller angle is caused by a modified gravitropic response of the mutant. In the *lazy1* mutant, the apical-basal polar auxin transport is increased, while lateral auxin transport is decreased. This results in abnormal auxin distribution leading to a weaker gravitropic response. Therefore, *LAZY1* controls gravitropism by regulating polar auxin transport (Li et al., 2007).

In conclusion, TAC1 and LAZY1 have opposite functions with respect to branch angle control (**Figure 2B**). The most obvious difference on the sequence level between TAC1 and LAZY1 is an EAR like domain at the C-terminus that is only present in LAZY1 (Dardick et al., 2013). Phylogenetic analyses and studies of intron–exon structure indicate that *LAZY1* is, from an evolutionary perspective, the older gene and *TAC1* evolved from *LAZY1* (Dardick et al., 2013). The opposing activities of these transcription factors may be explained by affinity of TAC1 and LAZY1 for the same promoter motifs. LAZY1 likely acts as a repressor through the EAR domain. TAC1, which lacks the EAR domain, may compete with LAZY1 and diminish repression by LAZY1 (Dardick et al., 2013).

Other genes that regulate tiller angle are *PROSTRATE GROWTH1* (*PROG1*; Tan et al., 2008) and *LOOSE PLANT ARCHITECTURE 1* (*LPA1*; Wu et al., 2013). Both genes encode putative zinc finger transcription factors with C-terminal EAR-like repression domains. The tiller base in the *prog1* mutant shows asymmetric growth due to a higher cell number on the lower side of the tiller base. Like *lazy1*, the *lpa1* mutant exhibits reduced shoot gravitropism, possibly caused by a slower sedimentation of amyloplasts in the statocytes (Wu et al., 2013).

In summary, the analyses on *TB1*, *TAC1*, *LAZY1, PROG1*, and *LPA1* in crop plants indicate that with respect to plant architecture, only few key genes have been the target of selection during domestication.

In the studies mentioned above, monocots were investigated. However, *TAC1* has also been identified as a candidate gene for branch angle control in dicotyledonous species, e.g., in peach trees (*Prunus persica*; Dardick et al., 2013). In trees, fruit and wood production are influenced by crown architecture. Trees with compact crowns are suited for high density cultivation andand allow yield increases compared to lines with a wider crown (Dardick et al., 2013). *P. persica* varieties that exhibit a compact growth habit are called broomy or pillar lines and the associated semidominant mutation has been designated as *br*. The mutation was mapped as an insertion that introduces a premature stop codon in a gene encoding a protein with similarity to the monocot TAC1. A knockout of the orthologous gene in *Arabidopsis* resulted in smaller angles between cauline (i.e., inflorescence) branches and the main inflorescence shoot as well as between rosette branches and the stem. The pyramid poplar (*Populus nigra* ‘Italica’) develops a phenotype comparable to the broomy or pillar variety of peach. This poplar growth habit may also be caused by a defect in a poplar ortholog of *TAC1*. In apple, another compact growth phenotype exists which has been designated columnar (*co*). However, this phenotype is different from the *P. persica* broomy or pillar growth habit. Columnar apple is not only characterized by a compact crown, but also by shorter branches, a thicker stem with shorter internodes and short fruit spurs (Petersen and Krost, 2013). Moreover, the *br* mutation is semidominant, while *co* is dominant. The *co* mutation has been mapped to a region of 393 kb with 36 ORFs on chromosome 10 (Petersen and Krost, 2013). However, the exact locus and its molecular function remain to be determined.

In fruit and timber trees, not only the branch angle, but also the degree of branching is economically important. The leaves of branches contribute to the specific leaf area index which significantly affects photosynthesis rate (Broeckx et al., 2012). In contrast to annual plants, trees build two different types of branches. During the growth period, the shoot apex suppresses the outgrowth of buds to a certain extent (apical dominance), leading to so-called paradormancy. However, this state of dormancy can be overcome by several factors (e.g., by decapitation), leading to bud outgrowth. Buds that develop and grow out in the same season without an intervening dormant season form so-called sylleptic branches. However, many species in temperate regions undergo dormancy during winter as an adaptation to adverse environmental conditions. After the growth period in summer, short day length and low temperatures prohibit further growth. The resulting stage of dormancy is called ecodormancy. It can still be broken if the growth conditions become more favorable. However, after further exposure to short daylength and low temperatures, the tree enters a stage called endodormancy, in which it can survive the harsh conditions in winter. Endodormancy can only be broken after a certain chilling requirement, i.e., a certain cumulative time of cold temperatures, is fulfilled. The plant is then reverted into an ecodormancy state, which will be broken when the environmental conditions become more favorable in spring. Buds formed during the previous growth period will then grow out and produce so-called proleptic branches. The different stages of dormancy described above are reviewed in Allona et al. (2008). Many tree species of the temperate regions form exclusively proleptic branches, but some genera like *Populus*, *Prunus*, *Alnus, Larix*, and *Tsuga* can also grow sylleptic branches (Broeckx et al., 2012). This may be advantageous during the establishment phase of trees since all branches that are built during the first growth period are, by definition, sylleptic branches. The additional leaf area of sylleptic branches contributes to carbon fixation and sylleptic branches have a high translocation efficiency of photosynthates (Scarascia-Mugnozza et al., 1999). Early canopy closure and the resulting suppression of weed growth might also be an important trait for fast growing trees on short rotation plantations.

In perennial plants, apical dominance seems to be controlled in a similar way as in annuals. Studies by Cline and Dong-Il (2002) indicate that auxin is a key player in this process. They compared three poplar clones with significant differences in sylleptic branching. They showed that “branchiness” of the three poplar clones correlates with sensitivity to auxin, which generally suppresses bud outgrowth: the clone with a low degree of sylleptic branching was more sensitive to auxin than the highly branched clone. A hallmark of branching control by apical dominance is a gradient of bud outgrowth across the main shoot. This is most evident and has been thoroughly characterized in *Arabidopsis*. Before flowering, bud initiation and outgrowth occurs in an acropetal direction while after the onset of flowering, this gradient is reversed and uppermost buds elongate and grow out first (Hempel and Feldman, 1994). Similarly, sylleptic branching occurs in a basipetal direction in poplar. In contrast, all proleptic branches that form after a period of dormancy start to elongate at a similar time point (Wilson, 2000). This synchronized growth of proleptic branches points to a control mechanism that is different from apical dominance or it is due to a factor that very efficiently breaks apical dominance. Studies by Moreno-Cortes et al. (2012) identified a protein that may play a role in bud outgrowth control. They isolated *CsRAV1* from chestnut that encodes a protein with homology to *AtRAV1* from *Arabidopsis* that has been classified as a transcriptional repressor (Ikeda and Ohme-Takagi, 2009). Overexpression of *CsRAV1* in poplar induced a high degree of sylleptic branching. Since the poplar clone that was used in these studies usually does not branch during the first growth period (i.e., it does not form sylleptic branches), suppression of branching must have been released by constitutive overexpression of CsRAV1. Interestingly, *CsRAV1* is highly expressed during winter. Moreno-Cortes et al. (2012) hypothesized that in perennials that grow in temperate regions, RAV1 accumulates during winter and elicits growth of proleptic branches from axillary meristems in the following spring. Overexpression of *RAV1*, thus, leads to season-independent accumulation of RAV1 and causes growth of sylleptic branches from meristems which have not been exposed to a period of winter dormancy.

## Conclusion

Apical dominance as a key control mechanism of branching has been a focus of intense research since Thimann and Skoog performed experiments in the 1930s on the role of auxin in suppression of branching (Thimann and Skoog, 1933). As it became evident that auxin does not directly suppress bud outgrowth, the second messenger hypothesis was put forward and the search for the elusive branching hormones initiated. Cytokinin was soon classified as one of the second messengers (Turnbull et al., 1997; Müller and Leyser, 2011), but it took until 2008 to identify SL as another branching hormone (Gomez-Roldan et al., 2008; Umehara et al., 2008). Within 6 years after this discovery, canonical SL biosynthesis and signaling pathways were established (Waldie et al., 2014). Now, SLs are accepted as branching control factors for herbaceous monocots (Umehara et al., 2008) and dicots (Gomez-Roldan et al., 2008). Loss-of-function mutants of SL biosynthesis and signaling show profound changes of plant architecture. Nonetheless, modification of the SL pathway has not yet been used in genetic engineering to improve architecture of crop plants. Also, SL genes have not been a target during monocot domestication, since the architectural trait selected during domestication of crops is low branching (Doust, 2007). However, the identification of *SLB1* and *SLB2* in rice cultivars (see discussion above) points to a role of SLs in parasitic weed resistance. Therefore, SLs may be an important target in breeding programs.

Analyses of domestication genes in monocot crops led to the identification of a small set of target genes (Doebley et al., 1997; Doust, 2007; Yu et al., 2007; Tan et al., 2008; Ku et al., 2011; Remigereau et al., 2011), of which each profoundly influences plant architecture. Originally, researchers proposed that monocot genes controlling plant architecture, e.g., *TB1* and *TAC1*, are unique to monocots (Doebley et al., 1997; Yu et al., 2007). However, orthologs of *TB1* and *TAC1* were soon also identified in dicots (Aguilar-Martinez et al., 2007; Martin-Trillo et al., 2011; Braun et al., 2012; Dardick et al., 2013). Now, with this knowledge, key genes for genetic engineering or for use as genetic markers for classical breeding of monocot and dicot crops are available.

In contrast to herbaceous plants, knowledge on branching control in woody plants generally is scarce. Currently, this topic is attracting more attention. Recent studies by Ward et al. (2013) and Czarnecki et al. (2014) showed complementation of *Arabidopsis max* mutants by *Salix* and *Populus MAX* orthologs, respectively, pointing to a role of SLs in trees. Also, *PpeTAC1* has been characterized as a controlling factor of branch angle in peach (Dardick et al., 2013) and *CsRAV1* from chestnut has been shown to play a role in seasonal control of proleptic branching (Moreno-Cortes et al., 2012).

Tree breeding is time consuming due to the long generation time of woody plants. Thus, using these key genes in genetic engineering approaches would be more straightforward to improve productivity. However, transgenic crops and also transgenic trees are not readily accepted by the public in many countries (Kaiser, 2001). Therefore, the generation of transgenic tree cultivars for wood or fruit production appears to be not economically reasonable at the moment. Alternatively, markers like *broomy* could be employed to assist classical breeding programs. Another powerful technique is Targeting Induced Local Lesions in Genomes (TILLING), which can identify desired point mutations in a mutagenized population in an efficient, high-throughput way (McCallum et al., 2000). A variant of this technique, called Ecotilling (Comai et al., 2004), could be used to screen natural populations for desired polymorphisms in order to exploit natural variation for breeding. These methods work without the production of genetically modified organisms (GMOs).

Additionally, targeted genome editing approaches such as CRISPR-Cas9 and related technologies may be used to actively introduce highly specific changes in the genome instead of screening for random changes (reviewed in Sander and Joung, 2014). However, it is still unclear how this and other new methods will be treated by legislature. Although, the resulting engineered plants cannot be distinguished from plants generated by traditional breeding methods, they may be classified as GMOs at least in the European Union, because their production involves transgenic intermediates (reviewed in Hartung and Schiemann, 2014).

Furthermore, even though the techniques discussed above are very powerful and may not fall under GMO-regulation, they are still limited to modifications of existing sequence within a given species. The introduction of entirely new sequences, allowing the attainment of completely new traits, can only be achieved by introducing foreign DNA, inevitably resulting in GMO by definition. Therefore, transgenic plants are still not entirely dispensable to match the demand for efficient crops and will most likely play a major role in the future in many countries.

## Conflict of Interest Statement

The authors declare that the research was conducted in the absence of any commercial or financial relationships that could be construed as a potential conflict of interest.

## Acknowledgment

We acknowledge funding by the German Ministry of Education and Research (FKZ 0315972C).
